# Improvement in Regional CBF by L-Serine Contributes to Its Neuroprotective Effect in Rats after Focal Cerebral Ischemia

**DOI:** 10.1371/journal.pone.0067044

**Published:** 2013-06-25

**Authors:** Tao-Jie Ren, Ren Qiang, Zheng-Lin Jiang, Guo-Hua Wang, Li Sun, Rui Jiang, Guang-Wei Zhao, Le-Yang Han

**Affiliations:** 1 Department of Neuropharmacology, Institute of Nautical Medicine, Nantong University, Nantong, Jiangsu Province, China; 2 Department of Infectious Diseases, The Third People’s Hospital, Nantong, Jiangsu Province, China; 3 Department of Neurology, Affiliated Hospital, Nantong University, Nantong, Jiangsu Province, China; 4 Department of Neurology, The First People’s Hospital, Yancheng, Jiangsu Province, China; Maastricht University, The Netherlands

## Abstract

To investigate the mechanisms underlying the neuroprotective effect of L-serine, permanent focal cerebral ischemia was induced by occlusion of the middle cerebral artery while monitoring cerebral blood flow (CBF). Rats were divided into control and L-serine-treated groups after middle cerebral artery occlusion. The neurological deficit score and brain infarct volume were assessed. Nissl staining was used to quantify the cortical injury. L-serine and D-serine levels in the ischemic cortex were analyzed with high performance liquid chromatography. We found that L-serine treatment: 1) reduced the neurological deficit score, infarct volume and cortical neuron loss in a dose-dependent manner; 2) improved CBF in the cortex, and this effect was inhibited in the presence of apamin plus charybdotoxin while the alleviation of both neurological deficit score and infarct volume was blocked; and 3) increased the amount of L-serine and D-serine in the cortex, and inhibition of the conversion of L-serine into D-serine by aminooxyacetic acid did not affect the reduction of neurological deficit score and infarct volume by L-serine. In conclusion, improvement in regional CBF by L-serine may contribute to its neuroprotective effect on the ischemic brain, potentially through vasodilation which is mediated by the small- and intermediate-conductance Ca^2+^-activated K^+^ channels on the cerebral blood vessel endothelium.

## Introduction

L-serine, a non-essential amino acid, plays a critical role in neuronal development and function in the central nervous system. As well as a building block of proteins, L-serine is a crucial neurotrophic factor and a precursor for phosphatidyl-L-serine, L-cysteine, nucleotides, sphingolipids, and neurotransmitters such as D-serine and glycine [1]. L-serine treatment has been used to manage depression, schizophrenia, and chronic fatigue syndrome, and to prevent psychomotor retardation, microcephaly, and seizures found in people with rare congenital defects of L-serine biosynthesis [1]. Interestingly, our previous study showed that L-serine exerted a neuroprotective effect that was potentially mediated by activating glycine receptors in the ischemic-reperfused brain of rats [2].

L-serine has also been shown to induce a reduction in mean arterial pressure via endothelium-dependent vasodilatation through the activation of apamin and charybdotoxin-sensitive Ca^2+^-activated K^+^ (K_Ca_) channels present on the endothelium [3], [4]. Although, this vasodilating action of L-serine has not been identified in cerebral blood vessels. However, we recently found an elevating action of L-serine on cerebral blood flow (CBF) in the rat after permanent focal cerebral ischemia. The present study was carried out to clarify whether the elevation of regional CBF (rCBF) by L-serine is mediated through the activation of apamin- and charybdotoxin-sensitive K_Ca_ channels on the endothelium and subsequent dilation of cerebral blood vessels. We also examined whether this vasodilating action would contribute to its neuroprotective effect on the brain after permanent middle cerebral artery occlusion (pMCAO) in rats.

## Materials and Methods

### Animals and Chemicals

Two hundred and eighty six male Sprague-Dawley rats weighting 270±10 g were used in the present study (obtained from the Experimental Animal Center of Nantong University, Nantong, China). All procedures were in accordance with the institutional guidelines of Nantong University, which comply with international rules and policies. Ethics in accordance with the ARRIVE guidelines were followed in animal experiments and approved by the Animal Care and Use Committee of Nantong University, Nantong, China.

L-serine, D-serine, charybdotoxin (ChTx), orthophthalaldehyde (OPA), aminooxyacetic acid (AOAA), apamin and strychnine hydrochloride were purchased from Sigma-Aldrich Corporation (Saint Louis, MO, USA); 2,3,5-triphenyltetrazolium chloride (TTC), methanol and N-acetyl-L-cysteine (NAC) from Merck Corporation (Darmstadt, Germany); and dimethylsulfoxide (DMSO) from Shenhe Chemical Reagent Co. Ltd. (Shanghai, China). All other chemicals were from Sinopharm Chemical Reagent Co. Ltd. (Shanghai, China) or Xilong Chemical Co. Ltd. (Guangzhou, China).

### Drug Administration

L-Serine was dissolved in normal saline, and used intraperitoneally (i.p.) at an interval of 12 h for 3 days. To determine the dose-dependent neuroprotective effect of L-serine, rats were randomly divided into 5 groups: sham-operated, vehicle, and L-serine 56 mg/kg, 168 mg/kg and 504 mg/kg. L-serine was initially used at 3 h after pMCAO. The vehicle group was treated with isodose saline. To investigate the time-window of L-serine efficacy, rats were randomly divided into 6 groups: vehicle and 5 L-serine treated groups. Onset of L-serine use (168 mg/kg, i.p.) was respectively at 1 h, 3 h, 6 h, 12 h and 24 h after pMCAO.

To determine the underlying mechanisms of the neuroprotective effect exerted by L-serine on the ischemic brain, L-serine 168 mg/kg was intraperitoneally used 3 h after pMCAO. A combination of apamin and charybdotoxin (75 µg/kg of each) were slowly infused through the internal jugular vein over a 10-min period and 45 min before the L-serine injection to ensure that the small- and intermediate-conductance K_Ca_ (SK_Ca_ and IK_Ca_) channels were blocked. In addition, strychnine (0.42 mg/kg, i.p.), or DMSO as a control, were used 5 min ahead of L-serine. Strychnine was dissolved with saline containing 0.1% DMSO.

For the measurement of D-serine and L-serine concentrations, L-serine 168 mg/kg was used intraperitoneally 3 h after pMCAO, and the samples were obtained at different time points: 0 h (control group, without use of L-serine), 0.5 h, 1 h, 2 h, 3 h, 6 h and 12 h after L-serine injection. For the observation of the influence of AOAA on L-serine efficacy, AOAA was intraperitoneally used at 25 mg/kg or 50 mg/kg 45 min ahead of the L-serine injection.

### MCAO Surgery

Rats were fasted overnight with free access to water. All animals were anesthetized first with 2 mL enflurane and anesthetization was maintained with 10% chloral hydrate (400 mg/kg, i.p.). Permanent MCAO was induced as reported previously [5]. Briefly, after a midline neck incision, the right common carotid artery (CCA), internal carotid artery (ICA) and external carotid artery (ECA) were exposed, and the proximal ECA and CCA were then ligated. The vagus nerve was carefully preserved as far as possible. A specialized nylon suture of 0.24 mm diameter (Beijing Biotech Co. Ltd., Beijing, China) was introduced from the lumen of the distal CCA just before bifurcation into the ICA until resistance was felt. Thus the origin of the middle cerebral artery (MCA) was occluded by the nylon suture. The average depth of filament insertion was 18.5±0.5 mm away from the bifurcation. Then, the exposed vessels were carefully ligated to prevent bleeding, and the incision was closed aseptically. Sham-operated animals were subjected to the same surgical procedure, but the suture was not advanced beyond the internal carotid bifurcation. A laser Doppler perfusion monitor (PeriFlux System 5010, Perimed, Stockholm, Sweden) was used to monitor rCBF throughout the study. The ischemic model was considered successful if about 75% reduction in CBF was induced immediately after placement of the suture [6], otherwise the animals were excluded. Rectal temperature of the rats was maintained at 37±0.5°C using a temperature-regulated heating pad throughout the anesthetic period including surgical preparation. After revival from anesthesia, the animals were put back into cages with the room temperature maintained at 25±2°C. Some of the rats died during the experiment, the mortality during the first 24 h after pMCAO operation was 18.8% and 16.9%, and the additional mortality until end-point was 5.5% and 5.0% respectively in the vehicle and L-serine treated groups. Data from these animals were excluded.

### Neurological Evaluation

Neurological function was evaluated at 72 h after pMCAO and scored on a 6-point scale [7]: 0, no neurological deficit; 1, failure to extend left forepaw fully; 2, circling to the left; 3, inability to bear weight on the left; 4, no spontaneous walking with depressed level of consciousness; and 5, death.

### Assessment of Infarct Size

Cerebral infarct size was assessed using the TTC staining method [8]. Animals were anesthetized with 10% chloral hydrate (500 mg/kg, i.p.) 72 h after pMCAO, and their brains were removed and sectioned into consecutive 2 mm-thick coronal slices using a mould (RBM-4000C, ASI Instruments, Warren, MI, USA). The slices were immediately immersed into 1% TTC medium at 37°C for 15 min and then turned over for another 15 min. The stained slices were washed in phosphate-buffered saline (PBS) for 5 min and then fixed in 4% buffered formaldehyde solution for 24 h. At the end of staining and fixation, color images of these slices were captured using a video camera (PowerShot S60, Canon, Tokyo, Japan). The infarct volume was analyzed using Image Pro Plus software. Percentage infarct volume was calculated as follows: [(V_C_–V_L_)/2V_C_]×100, where V_C_ is the volume of control hemisphere (left side), V_L_ the volume of non-infarcted tissue in the lesioned hemisphere (right side).

### Nissl Staining

72 h after pMCAO, rats were anesthetized with 10% chloral hydrate (500 mg/kg, i.p.) and perfused with 250 mL of normal saline and subsequently with 200 mL of 4% paraformaldehyde in 0.1 M PBS (pH 7.4). Rat brain was removed and then post-fixed for 24 h in the same fixative. The post-fixed brain tissue was cryo-protected in 20% then 30% sucrose in PBS. The brain tissue was then sectioned coronally 20 µm in thickness with a cryostat slicer (CM1900, Leica, Bensheim, Germany). The sections from bregma −2.60 mm to −3.2 mm were prepared, mounted with neutral balata (Shanghai Specimen and Model Factory, Shanghai, China) and blotted onto slides, and then covered with a coverslip after Nissl staining, which was performed as reported previously [2].

After Nissl staining, neuronal cells in the cortex of the infarct area were identified under a high-magnification (×400) light microscope and counted by an investigator blinded to the grouping. For each rat, the mean number of neurons was obtained by examining three serial coronal sections. In each section, the number of neurons was averaged from three different vision fields of the cortex. Only intact neurons with a clearly defined cell body and nucleus were counted.

### High-performance Liquid Chromatography (HPLC) Analysis

We used the method described by Kiya et al. [9]. Rats were anesthetized with 10% chloral hydrate (500 mg/kg). Blood drawn from right ventricle was dissolved in physiological saline containing 1.12 mg/mL ethylene diamine tetraacetic acid-2Na (EDTA-2Na), the volume of which was nine times as much as blood. The blood samples were then centrifuged at 15,000×g for 10 min at 4°C to obtain the plasma. At the same time, the ischemic cortex was rapidly harvested and frozen at −80°C together with the plasma. The cortex was homogenized in methanol on ice using an ultrasonic cell crusher. The volume (mL) of methanol added was equivalent to the weight (mg) of the brain. The plasma was diluted 99-fold in methanol. The brain homogenates and diluted plasma were then centrifuged at 15,000×g for 10 min at 4°C. The supernatants were mixed with isometric 5 mM OPA/NAC solution. After standing at room temperature for 2 min, 20 µl of the reaction mixture was injected into an HPLC system (Shimadzu, Kyoto, Japan). Samples and standard solutions were automatically derivatized and injected onto the HPLC system. Diastereomeric derivatives of D-serine and L-serine in OPA/NAC were separated on a C18 chromatographic column, and the fluorescence was monitored with an excitation wavelength of 340 nm and an emission wavelength of 445 nm. A mixture of 18% (v/v) methanol and 82% (v/v) 0.1M PBS (pH 6.0) containing 5 mg/L EDTA-2Na was used as the mobile phase. After elution of D- and L-serine derivatives, late eluting peaks were flushed out with a mixture of 80% methanol and 20% 0.05M PBS (pH 6.0) containing 5 mg/L EDTA-2Na. The mobile phase flow rate was set at 1 mL/min, and the column temperature was set at 30°C.

### Measurement of rCBF and Other Physiological Parameters

According to the literature [6], CBF and brain tissue oxygen saturation (SbtO_2_) were continuously monitored with a laser Doppler perfusion monitor (PeriFlux System 5000, Perimed) and the moorVMS-OXY™ system (Moor Instruments, Devon, UK). Briefly, a sagittal skin incision about 1.5 cm long was made. Scanning probes (407-1, Perimed; CP2T-1000, Moor Instruments) were carefully placed on the skull ipsilateral to the pMCAO under a stereotaxic device (51653, Stoelting Co., Wood Dale, IL, USA) with its center at 5 mm lateral to midline and 1 mm posterior to bregma, thus avoiding any large vessel. After successful operation, the rats were continually kept anesthetized to detect the influence of L-serine on CBF and SbtO_2_ in the ischemic cortex. The basal level of rCBF prior to pMCAO was assigned a value of 100%, and the rCBF levels under other conditions were calculated as percent values relative to the basal level. Mean values of SbtO_2_ were obtained by calculating the average level of 5 min monitoring before pMCAO, 30 min after pMCAO and 30 min after use of L-serine or vehicle.

To verify the change in rCBF detected with the laser Doppler perfusion monitor, cerebral perfusion imaging of rat brain was performed using computed tomograph (CT) perfusion imaging technique. The anesthetization of rats was maintained with 10% chloral hydrate (400 mg/kg, i.p.). L-serine (168 mg/kg) or the equal volume of vehicle (normal saline) was injected intraperitoneally 30 min after pMCAO. One hour later, non-enhanced CT scans were continuously carried out using the Philips Brilliance 64 CT scanner (Royal Philips Electronics, Amsterdam, Netherlands) with a bolus injection of 2 ml ioversol 320 through tail vein at a rate of 0.3 ml/s at the start of scan (slice thickness 2.5 mm, 80 kV, 100 mA, scan speed 1 slice/s). The layer with maximal ischemic area was selected for the comparison of CBF relative to the non-ischemic side of the brain. Relative CBF of the region of interest was calculated by the software of the CT system. Other three parameters of relative cerebral blood volume (CBV), mean transit time (MTT) and time to peak (TTP) were simultaneously obtained according to the literature [10], [11].

During the measurement of rCBF, transcutaneous partial pressure of O_2_ (tcPO_2_) was simultaneously monitored from the hip skin of the rat using a probe (E5250, Perimed) [12]. Blood pressure and heart rate were simultaneously measured with a caudal artery pressure measuring system (Alcott Biotech Co. Ltd., Shanghai, China).

### Statistics

Data are presented as the mean ± standard error. Neurological deficit score data were compared using the Kruskal-Wallis rank-sum test and the Extended t-test for post hoc comparisons. Data from multiple groups were analyzed using one-way ANOVA and Scheffé’s test for post hoc comparisons. Data of cerebral L-serine and D-serine content were analyzed using two-way ANOVA followed by one-way ANOVA for the same treatments, and Student’s t-test for two different treatments at the same time point. Differences with P values less than 0.05 were considered statistically significant.

## Results

### Monitoring Physiological Parameters

Blood pressure, heart rate and tcPO_2_ were monitored; however, no notable influence was observed after the use of L-serine, apamin plus ChTx, or a combination of L-serine and apamin plus ChTx (Table 1).

**Table 1:** Influence of L-serine on physiological parameters in pMCAO rats.

Group (n = 6)	HR (beat/min)	MAP (mmHg)	tcPO_2_ (mmHg)
Sham-operated	348±11	97.5±5.1	83.1±3.8
Vehicle	367±6	102.8±4.0	82.5±3.5
L-serine	361±7	99.4±4.2	85.4±3.2
Apamin+ChTx	357±8	98.9±5.7	84.3±3.7
Apamin+ChTx+L-serine	359±8	104.0±3.2	82.9±2.7

HR, heart rate; MAP, mean arterial pressure; pMCAO, permanent middle cerebral artery occlusion; tcPO_2_, transcutaneous partial pressure of O_2_; and ChTx, charybdotoxin.

### Dose-dependent Neuroprotective Effect of L-serine

**Figure 1:**
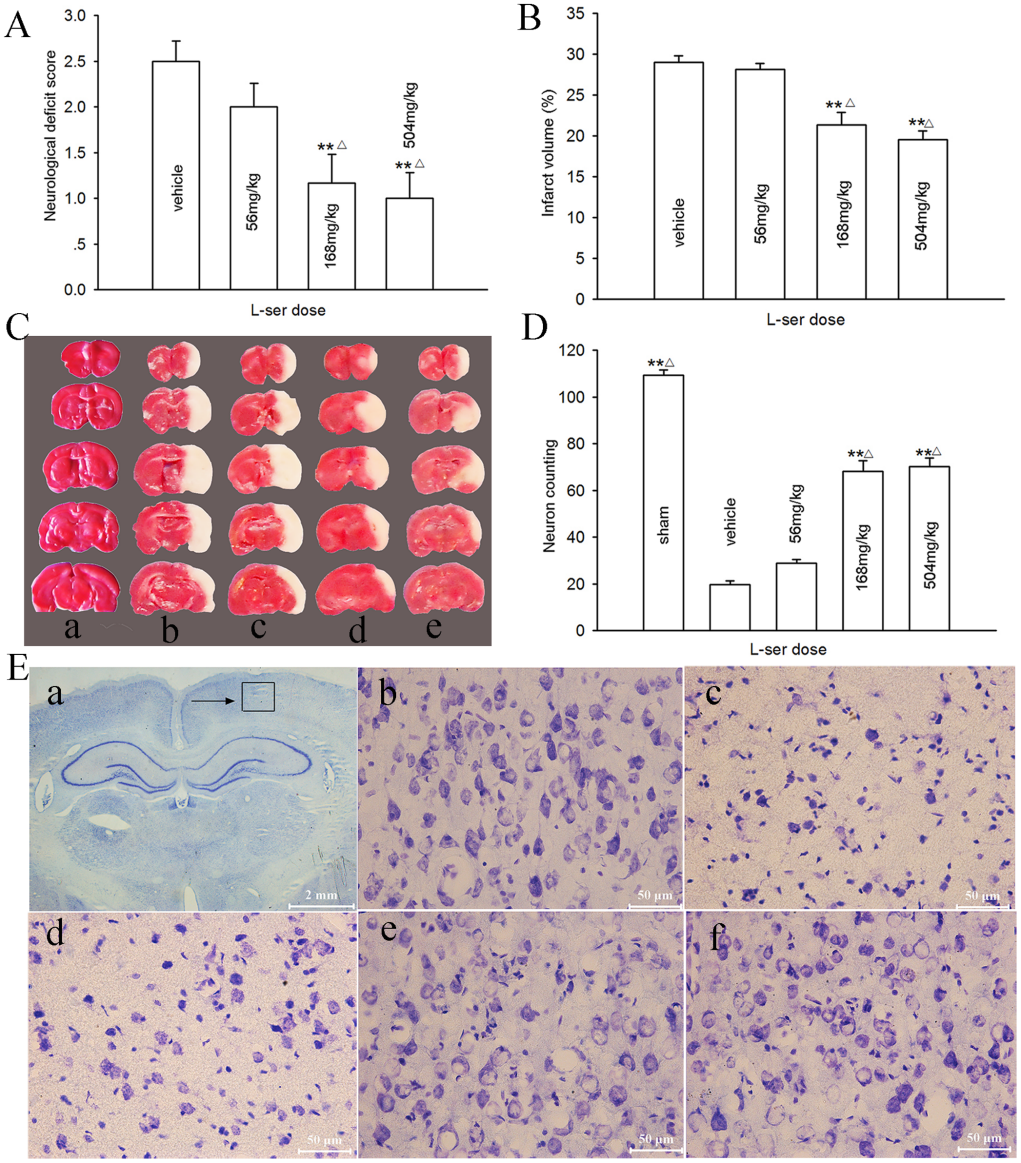
Dose-dependent neuroprotective effect of L-serine on the rat brain after pMCAO. L-serine was initially used at 3 h after pMCAO. (A) Neurological deficit score. (B) Infarct volume (six rats per group, main effect of L-serine concentration: *F*_1, 23_ = 29.83, *P = *0.000). (C) Examples of TTC staining after each treatment: a, sham-operated; b, vehicle; c, L-serine 56 mg/kg; d, L-serine 168 mg/kg; e, L-serine 504 mg/kg. (D) Number of normal neurons counted (four rats for each group, main effect of L-serine concentration: *F*_1, 19_ = 146.25, *P = *0.000). (E) Examples of Nissl staining: a, area of neurons counted (→); b, sham-operated; c, vehicle; d, L-serine 56 mg/kg; e, L-serine 168 mg/kg; f, L-serine 504 mg/kg. **P<0.01, vs. vehicle; ^△^ *P*<0.05, vs. L-serine 56 mg/kg group. L-ser, L-serine.

A dose-dependent neuroprotective effect of L-serine was examined in rats 72 h after pMCAO. The neurological function 72 h after pMCAO was reduced with an elevation in the neurological deficit score (Fig. 1A), and an infarct was induced with a volume of 410.6±8.6 mm^3^ (Fig. 1B–C). However, L-serine treatment alleviated the neurological deficit score and infarct volume in a dose-dependent manner: i.e. L-serine at 56 mg/kg had no affect (*p*>0.05); at 168 mg/kg, the neurological deficit score was significantly reduced by L-serine from the control value of 2.50±0.22 to 1.17±0.31 (*p*<0.01), and the infarct volume from 29.0±0.8% to 21.4±1.5% (*p*<0.01). A higher dose of L-serine (504 mg/kg) further reduced the neurological deficit score and infarct volume, but the reductions were not significantly different from those of the 168 mg/kg group (*p*>0.05, Fig. 1A–B).

Using Nissl staining, a severe neuronal loss in the parietal cortex ipsilateral to pMCAO was found (20±3.4 vs. 109±5.0 for sham-operated rats, Fig. 1D and E). L-Serine treatment after pMCAO significantly inhibited the decrease in neuronal number; the mean value was increased to 68±9.0 and 70±7.0 in the 168 mg/kg and 504 mg/kg groups respectively (*p*<0.01, Fig. 1D). No noticeable differences in the number of neurons were noted between these two doses (*p*>0.05, Fig. 1D). Therefore, we selected the 168 mg/kg dose for the following experiments.

### Time-window of L-serine Efficacy

**Figure 2:**
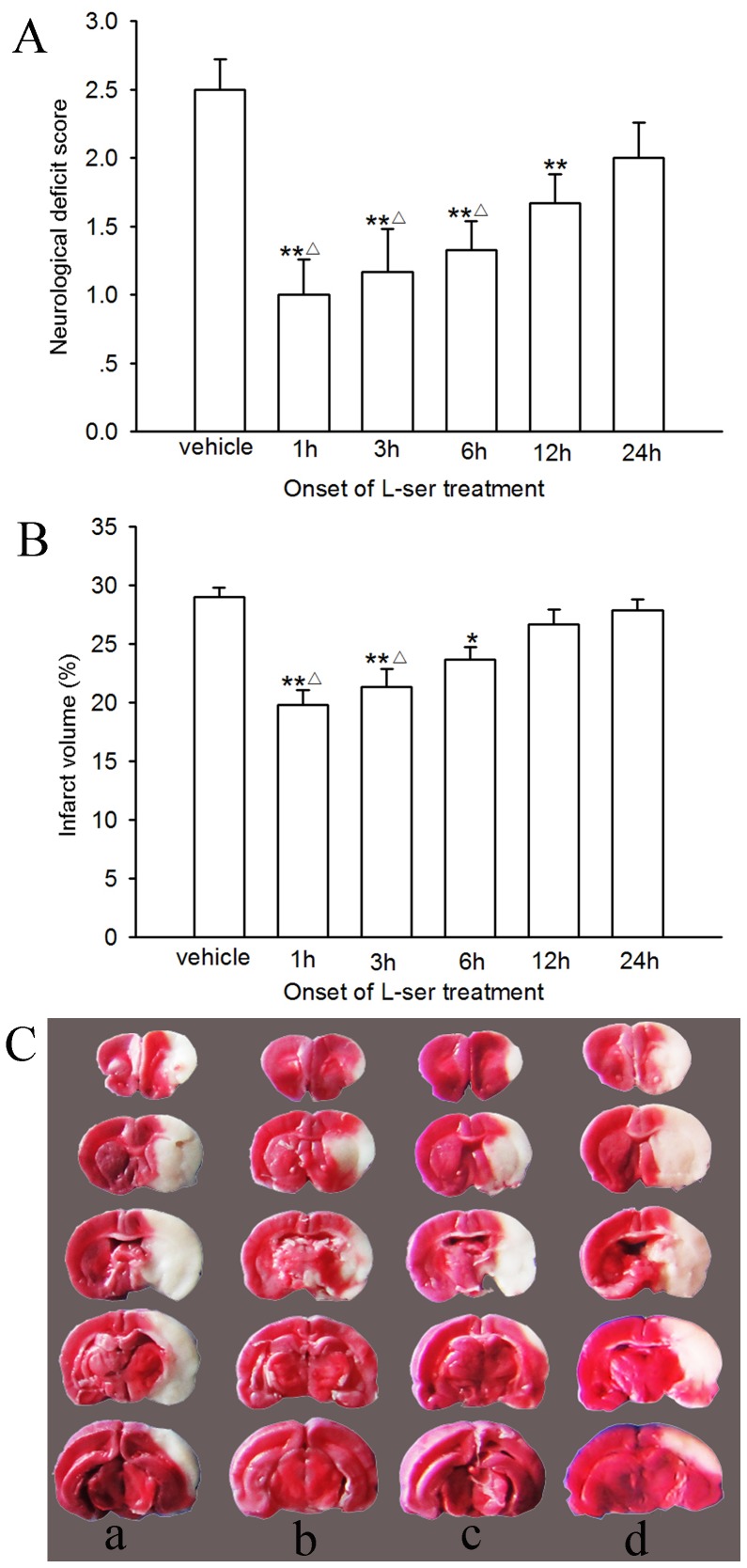
Time-window of L-serine efficacy in pMCAO rats (six rats per group). L-serine was used at 168 mg/kg. (A) Neurological deficit score. (B) Infarct volume (main effect of different time points: *F*_1, 35_ = 10.00, *P = *0.000). (C) Examples of TTC staining: a, vehicle; b, 1 h group; c, 3 h group; d, 12 h group. *P<0.05, **P<0.01, vs. vehicle; ^△^ *P*<0.05, vs. 24 h group. L-ser, L-serine.

To observe the time window of L-serine efficacy, L-serine (168 mg/kg, i.p.) was used at 1 h, 3 h, 6 h, 12 h or 24 h after pMCAO and repeated every 12 h for 3 days. The neurological function and infarct volume were determined 72 h after pMCAO. As shown in Fig. 2, first treatment of L-serine conducted at 1 h, 3 h or 6 h after pMCAO exerted a prominent neuroprotective effect in contrast to that at 12 or 24 h after pMCAO: i.e. first treatment of L-serine at 1 h, 3 h or 6 h after pMCAO decreased both the neurological deficit score and infarct volume notably (*p*<0.01 or 0.05), but that at 24 h after pMCAO did not (*p*>0.05). First use of L-serine at 12 h after pMCAO reduced only the neurological deficit score (*p*<0.01), but not the infarct volume (*p*>0.05, Fig. 2).

### Increase in rCBF by L-serine and the Underlying Mechanisms

**Figure 3:**
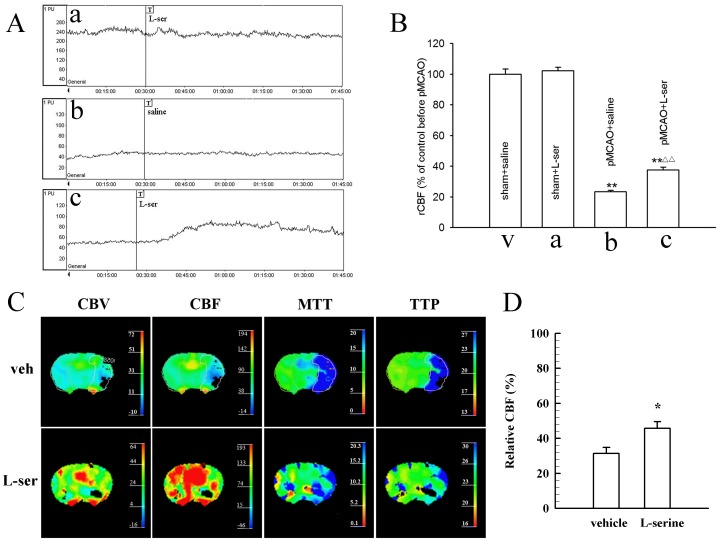
Elevation of rCBF after single intraperitoneal injection of L-serine. (A) Typical tracings of rCBF, where “T” in a, b and c indicates the onset time of different treatments: a, use of L-serine in sham-operated rat; b, vehicle group rat after pMCAO; c, use of L-serine in rat after pMCAO. (B) Mean values of rCBF in different groups (six rats per group, main effect of different treatments: *F*_1, 23_ = 11.74, *P = *0.000). **P<0.01, vs. sham groups; ^△△^ *P*<0.01, vs. pMCAO+saline group. L-ser, L-serine. (C) Examples of CT perfusion imaging after use of L-serine or vehicle. Enclosed was the region of interest and the same as in the L-serine treated rat. (D) Mean values of relative CBF after use of L-serine or vehicle (four rats for each group). Data of CBV, MTT and TTP were not shown. *P<0.05, vs. vehicle group (Student’s *t*-test was used for the analysis).

To investigate the potential mechanism of the neuroprotective effect exerted by L-serine, we measured the CBF of the cortex with the laser Doppler perfusion monitor. L-serine treatment (168 mg/kg) did not change CBF of the cortex ipsilateral to the ischemic side in the sham-operated rat (Fig. 3A–a), but elevated CBF of the ischemic cortex in the pMCAO rat (Fig. 3A–c); the mean value increased from 23.5±0.9% to 37.7±1.6% (*p*<0.01, Fig. 3B).

Similar increase in the rCBF of the ischemic side of rat brain after use of L-serine (168 mg/kg) was also detected by CT perfusion imaging technique (*p*<0.05, Fig. 3C and D). Moreover, the influence of L-serine on rCBF in a mouse pMCAO model was investigated using the laser speckle technique (see ). Likewise, intraperitoneal use of L-serine (268 mg/kg) increased rCBF of the ischemic brain of mice at 55 min after pMCAO (*p*<0.01, see Figure S1).

Similar to change in the rCBF, cortical SbtO_2_ was reduced markedly in the rat after pMCAO (Table 2). However, SbtO_2_ was increased by 16.1±2.97% after the use of L-serine (168 mg/kg, i.p., *p*<0.01, Table 2).

**Table 2:** Influence of L-serine on SbtO_2_ in pMCAO rats (%).

Group (n)	Basal level	30min after pMCAO	30min after use of L-serine or normal saline	Percent change
Vehicle (4)	55.5±0.59	25.5±1.93	25.0±2.70	−3.5±4.75
L-serine (7)	54.8±0.88	25.4±1.31	30.5±1.74	16.1±2.97**

Student’s *t*-test was used for the statistical analysis.

** *p*<0.01, vs. vehicle group. SbtO_2_, brain tissue oxygen saturation.

**Figure 4:**
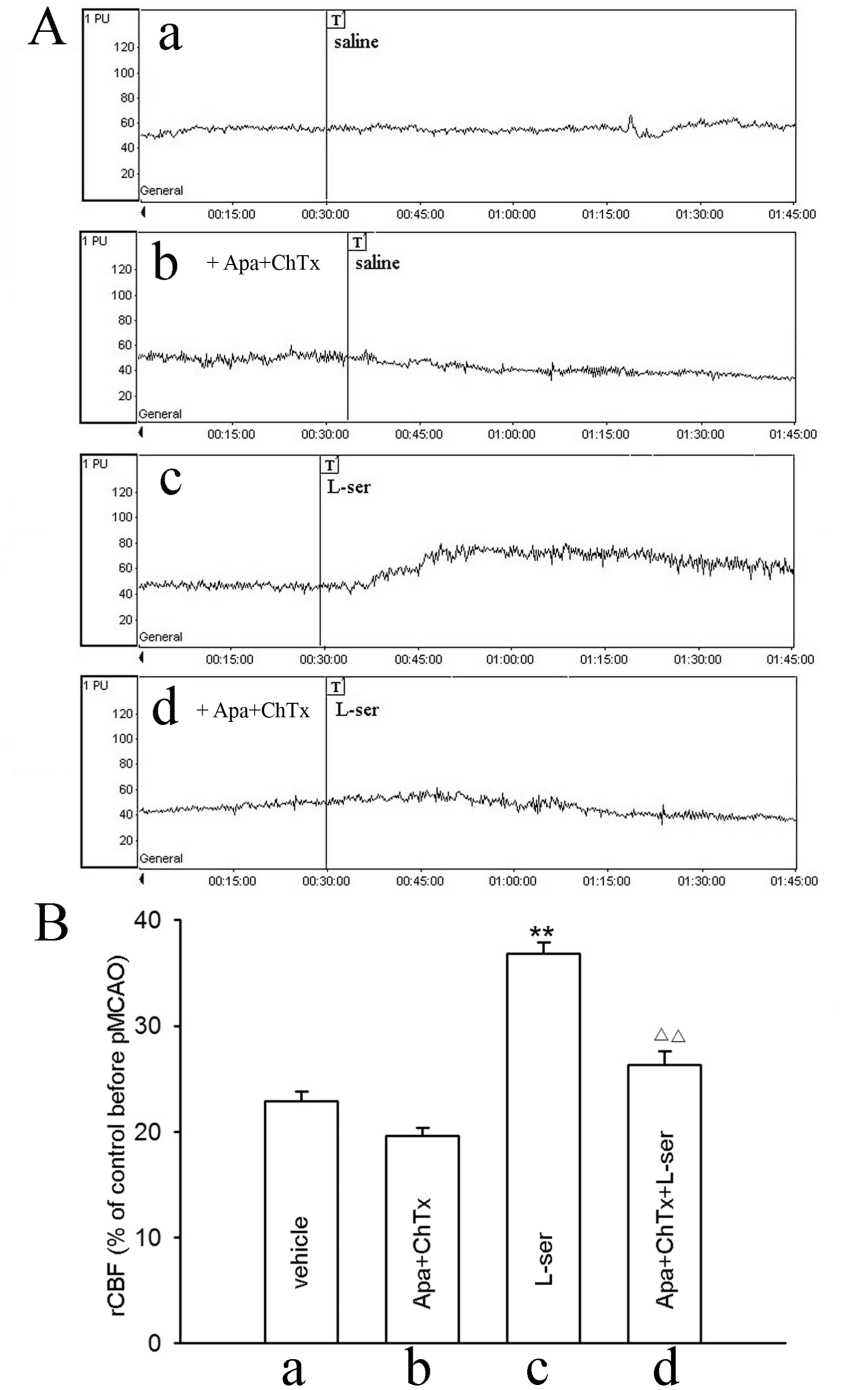
Influence of apamin plus ChTx on rCBF after use of L-serine in pMCAO rats (six rats per group). (A) Typical tracings of rCBF, where “T” in a, b, c and d indicates the onset time of different treatments: a, vehicle; b, addition of apamin plus ChTx in vehicle rat; c, use of L-serine; d, addition of apamin plus ChTx before use of L-serine. (B) rCBF in different groups (main effect of different treatments: *F*_1, 23_ = 51.66, *P = *0.000). **P<0.01, vs. vehicle group; ^△△^ *P*<0.01, vs. L-serine-treated group. L-ser, L-serine.

To investigate whether elevation of rCBF by L-serine was mediated through opening SK_Ca_ and IK_Ca_ channels on the vascular endothelium, we infused apamin plus ChTx (both 75 µg/kg) to block these channels *in vivo* [3], [4]. Intravenous use of apamin and ChTx did not exert significant influence on rCBF by themselves (Fig. 4A–b), but blocked the elevating effect of L-serine on rCBF (Fig. 4A–d). The mean value of rCBF was reduced from 36.8±1.0% in the L-serine group to 26.3±1.4% in the apamin+ChTx+L-serine group (*p*<0.01, Fig. 4B).

**Figure 5:**
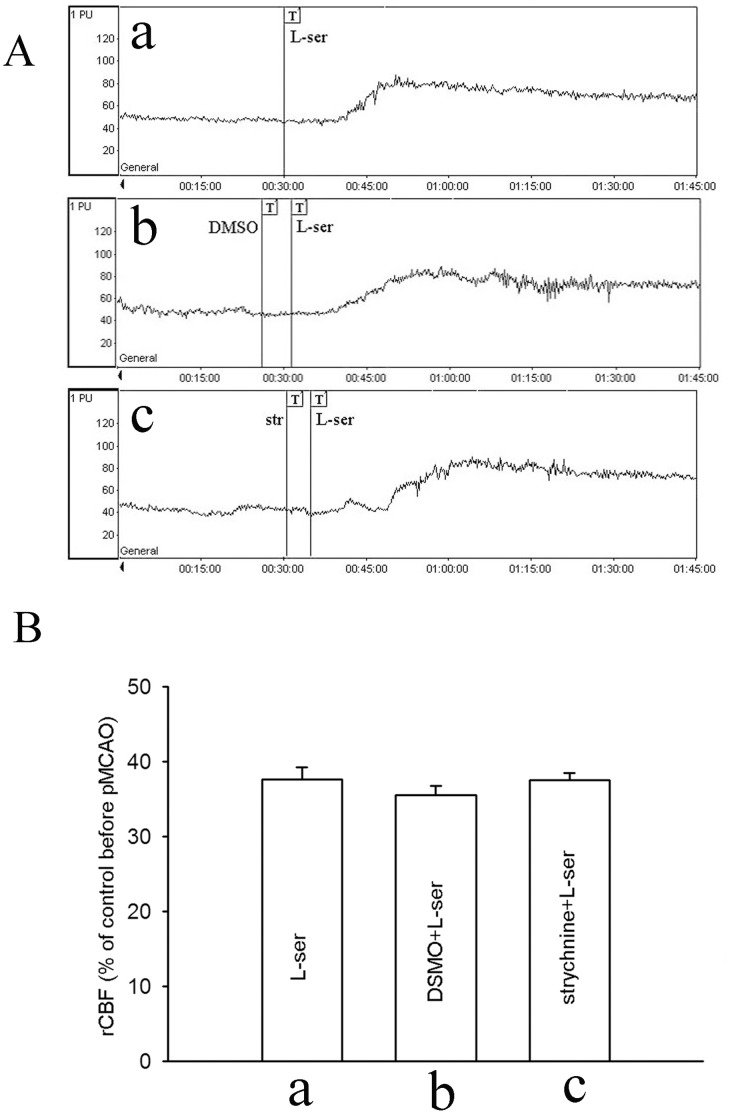
Influence of strychnine on the effect of L-serine treatment in rCBF (six rats per group). (A) Typical tracings of rCBF: a, use of L-serine after pMCAO; b, addition of DMSO (vehicle for strychnine, as control); c, addition of strychnine before use of L-serine. (B) rCBF in different groups (main effect of different treatments: *F*_1, 17_ = 0.87, *P = *0.442). L-ser, L-serine; str, strychnine.

To exclude the possibility that the effect of L-serine on rCBF was mediated through activating glycine receptors, we used strychnine (i.p.), a glycine receptor antagonist, at a subconvulsant dose of 0.42 mg/kg [13], [14]. As shown in Fig. 5A, strychnine did not exert any significant influence on the effect of L-serine. Mean values of rCBF in L-serine, DMSO and strychnine groups were 37.6±1.5%, 35.6±1.2% and 37.5±1.9%, respectively (*p*>0.05, Fig. 5B).

### Blunting of the Neuroprotective Effect of L-serine by Apamin Plus ChTx

**Figure 6:**
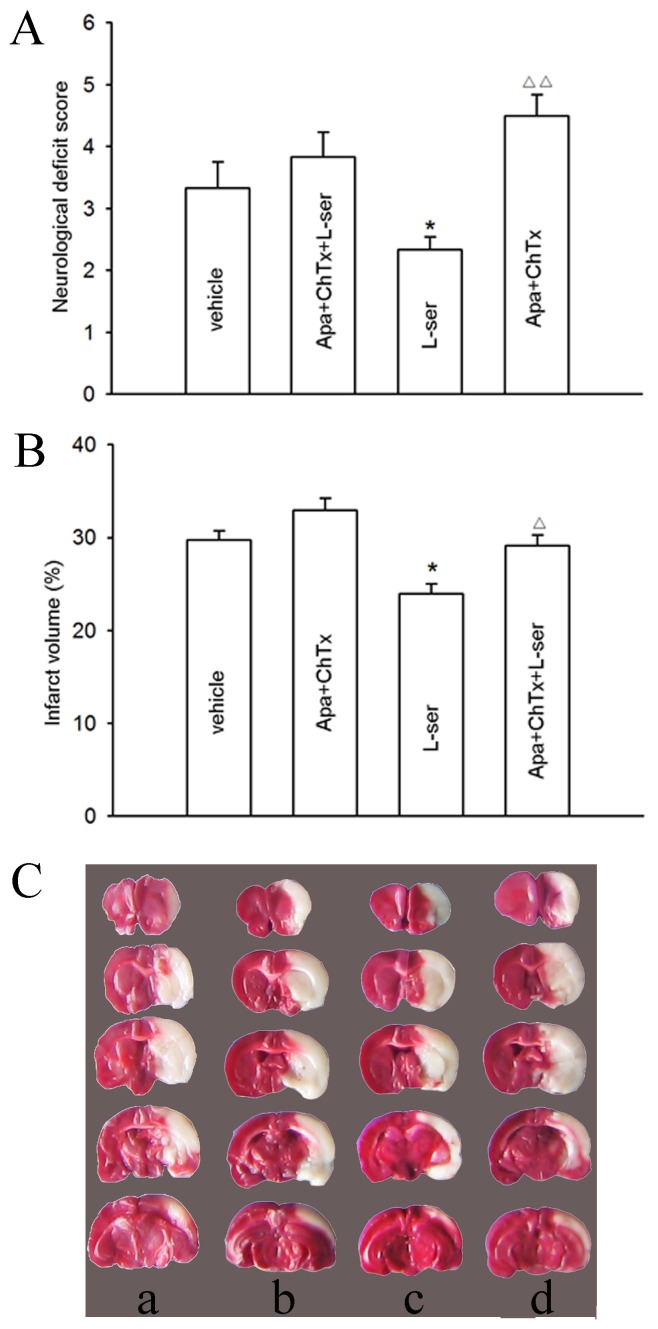
Influence of apamin plus ChTx on the neuroprotective effect of L-serine in pMCAO rats (six rats per group). (A) Neurological deficit score. (B) Infarct volume (main effect of different treatments: *F*_1, 23_ = 11.17, *P = *0.000). (C) Examples of TTC staining after different treatments: a, vehicle; b, addition of apamin plus ChTx in vehicle rat; c, use of L-serine; d, addition of apamin plus ChTx before use of L-serine. *P<0.05, vs. vehicle; ^△^ *P*<0.05, ^△△^ *P*<0.01, vs. L-serine-treated group. L-ser, L-serine.

To verify the mechanism underlying the neuroprotective effect of L-serine in rats, we carried out further studies using apamin and ChTx. The neurological deficit score in the apamin+ChTx group was a little higher than that of the vehicle group, but not significant (*p*>0.05, Fig. 6A). However, pretreatment of apamin and ChTx blunted the reducing effect of L-serine on the neurological deficit score (Fig. 6A). A similar result was detected for the infarct volume (Fig. 6B and 6C); i.e. in the presence of apamin and ChTx, the reducing effect of L-serine on the infarct volume was also blunted, the mean value of which was raised from 23.7±1.0% to 29.1±1.1% (*p*<0.05, Fig. 6B).

### Measurement of L-serine and D-serine Concentrations in the Brain

Ischemic rats were injected with L-serine (168 mg/kg, i.p.) 3 h after MCAO. Normal rats received the same treatment. Samples of blood and ischemic cortex were harvested at 0.5 h, 1 h, 2 h, 3 h, 6 h and 12 h after L-serine injection.

**Figure 7:**
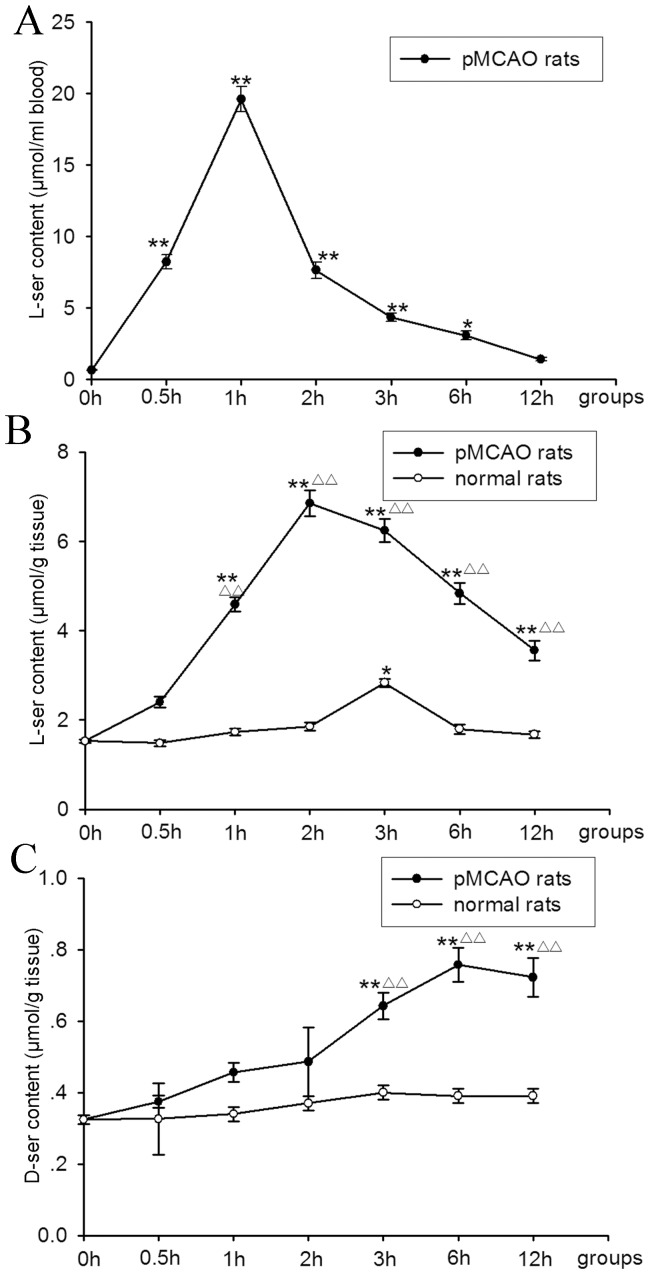
Concentration of L-serine and D-serine (six rats per group). (A) Concentration of L-serine in the blood (*F*_1, 41_ = 195.04, *P* = 0.000). (B) Concentration of L-serine in the cortex ipsilateral to pMCAO (different treatments: *F*_2, 1_ = 875.18, *P* = 0.000; different time points: *F*_2, 6_ = 104.91, *P* = 0.000). (C) Concentration of D-serine in the cortex ipsilateral to pMCAO (different treatments: *F*_2, 1_ = 124.96, *P* = 0.000; different time points: *F*_2, 6_ = 24.35, *P* = 0.000). *P<0.05, **P<0.01, vs. 0 h group (without use of L-serine); ^△△^ *P*<0.01, vs. the normal rats at the corresponding time point. L-ser, L-serine; D-ser, D-serine.

As shown in Fig. 7A, after L-serine treatment, the concentration of L-serine in the blood raised rapidly; the peak level (19.59±0.88 µmol/mL) appeared at 1 h after L-serine injection (*p*<0.01), and then the concentration of L-serine decreased with time.

The concentration of L-serine in the ischemic cortex significant increased, similar to that seen in blood. After 1-h delay, the peak level was reached at 2 h after L-serine injection, being several times more than the vehicle group (0 h) (6.85±0.30 vs. 1.52±0.04 µmol/g, *p*<0.01, Fig. 7B). The L-serine level at 12 h after injection (3.56±0.22 µmol/g) was still significantly higher than that of the vehicle group (*p*<0.01). However, L-serine levels in the brain of normal rats increased slowly; the peak level was reached at 3 h after L-serine injection (2.83±0.09 µmol/g, *p*<0.05), and then the concentration of L-serine decreased rapidly to the basal level (Fig. 7B).

Moreover, the concentration of D-serine in the ischemic cortex was also increased after L-serine treatment, but in a different pattern (Fig. 7C); i.e. D-serine level increased gradually with a peak concentration at 6 h after L-serine injection (0.75±0.04 vs. 0.32±0.01 µmol/g of vehicle group, *p*<0.01). However, the D-serine level in normal rats was not markedly increased after L-serine treatment (*p*>0.05, Fig. 7C).

### Influence of AOAA on the Neuroprotective Effect of L-serine

**Figure 8:**
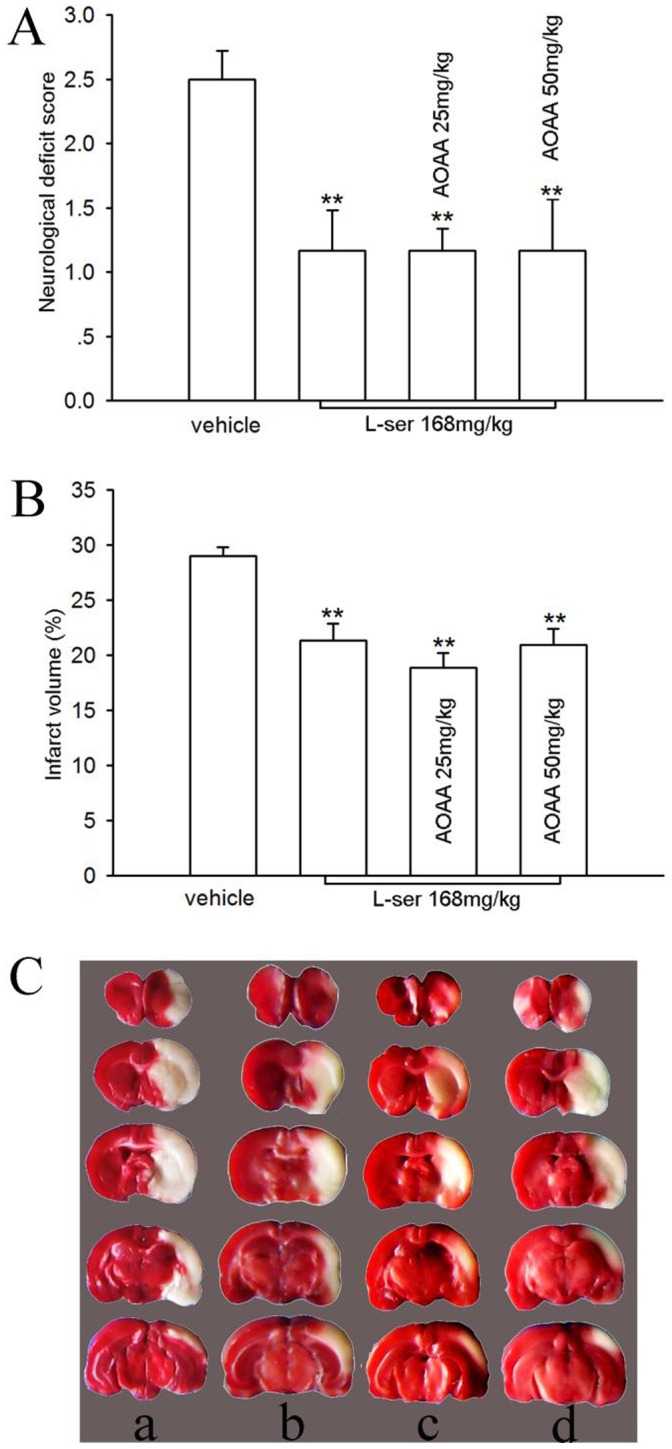
Influence of AOAA on the neuroprotective effect of L-serine in pMCAO rats (six rats per group). (A) Neurological deficit score. (B) Infarct volume (main effect of different treatments: *F*_1, 23_ = 11.74, *P = *0.000). (C) Examples of TTC staining after different treatments: a, vehicle; b, L-serine; c, L-serine+AOAA 25 mg/kg; d, L-serine+AOAA 50 mg/kg. **P<0.01, vs. vehicle. L-ser, L-serine.

It has been reported that pyridoxal 5′-phosphate-dependent serine racemase converts L-serine into D-serine [15] which is a potent *N*-methyl-D-aspartate (NMDA) receptor co-agonist [16]. AOAA is a potent inhibitor of pyridoxal phosphate-dependent enzymes [17]. Because the above HPLC results showed that L-serine was partly converted into D-serine, AOAA was used ahead of the L-serine injection to see whether converted D-serine would affect the neuroprotective effect of L-serine. Nevertheless, the effects of L-serine on both the neurological deficit score and infarct volume were not altered significantly by AOAA (*p*>0.05, Fig. 8A–C).

## Discussion

The present study found that L-serine used after pMCAO reduced the neurological deficit, infarct volume and cortical neuronal loss in a dose- and time-dependent manner, suggesting that L-serine can exert a neuroprotective effect on the brain in permanent cerebral ischemic rats. These results extend and are consistent with our previous observation on the effect of L-serine in ischemic/reperfused rat after MCAO [2].

To investigate the mechanisms underlying the neuroprotective effect of L-serine, rCBF was monitored before and after pMCAO and after use of L-serine. Surprisingly, we found that L-serine did not influence CBF of normal rats, but improved CBF and oxygen saturation of the ischemic cortex in pMCAO rats. This increase in the CBF of ischemic brain after L-serine treatment was also detected in the pMCAO mice. We suggest that the neuroprotective effect of L-serine may be mediated, at least partly, by improving CBF of the ischemic brain, a finding which has not been reported previously. This is a novel mechanism of the neuroprotective effect of L-serine apart from the glycine receptor-mediated mechanism we reported previously [2].

Because glycine receptors were involved in mediating the neuroprotective effect of L-serine, as we reported previously [2], strychnine, an antagonist of glycine receptors, was applied before using L-serine. The elevating effect of L-serine on rCBF was not influenced by strychnine, suggesting that the effect of L-serine on rCBF is not mediated by glycine receptors.

The vascular endothelium controls vessel tone by releasing nitric oxide (NO) and prostacyclin, as well as by a third pathway involving endothelium-derived hyperpolarizing factors (EDHF) [16]. EDHF-mediated processes involve an increase in the intracellular calcium concentration, opening of the endothelial SK_Ca_ and IK_Ca_ channels and hyperpolarization of the endothelial cells [18]. The role of EDHF is negligible in the aorta, accounts for about half of the total vasodilatory response in carotid arteries with a diameter less than 500 µm, and seems to be predominant in arterioles (<100 µm) [18], [19]. McNeish et al. have confirmed SK_Ca_ and IK_Ca_ channels present on the endothelium of the rat MCA, and both IK_Ca_ and SK_Ca_ channels are involved in the hyperpolarizing responses of the rat MCA [20]. Cipolla et al. have found that SK_Ca_ and IK_Ca_ channel activity could diminish the basal tone of the cerebral parenchymal arterioles (PA), but not MCA, and the EDHF responsiveness of PA after ischemia/reperfusion may be preserved and have an important role in vasodilation under the conditions when NO synthase has been inhibited [19]. Moreover, Hannah et al. have reported that both SK_Ca_ and IK_Ca_ channels conduct the outward current in isolated PA endothelial cells, and blockade of SK_Ca_ and IK_Ca_ channels in PA will decrease the resting cortical CBF by ∼15% while opening the channels increased CBF by ∼40% [21]. Similarly, Mishra et al. have found that L-serine could evoke a rapid, reversible, dose-dependent vasodilatation of the third-order branches of mesenteric arterioles by activating SK_Ca_ and IK_Ca_ channels without increasing the heart rate, and this effect is more pronounced in NO synthase-blunted rats than in control rats [3]. In areas where ischemic CBF is <30% of pre-occlusion values, auto-regulation is completely lost, but the EDHF-mediated mechanism continues to function [22]. We did not detect any significant effect of L-serine on CBF in normal rats, possibly because normal auto-regulation keeps CBF constant in non-ischemic rats.

For these reasons, we supposed that L-serine might improve CBF in the ischemic area through opening SK_Ca_ and IK_Ca_ channels. In order to confirm this, we carried out further studies. According to the method described by Mishra et al. [3], [4], we used apamin and ChTx to block both SK_Ca_ and IK_Ca_ channels. Surprisingly, the effect of L-serine enhancing blood flow in the ischemic cortex was blocked, suggesting that L-serine may induce vasodilation in ischemic brain tissue to elevate rCBF through SK_Ca_ and IK_Ca_ channels on the endothelial cells of cerebral blood vessels.

To clarify whether this vasodilating action contributes to the neuroprotective effect of L-serine, an additional experiment was carried out. With pretreatment of SK_Ca_ and IK_Ca_ channels blockers, the reducing effect of L-serine on the neurological deficit score and infarct volume was significantly reduced, suggesting that the neuroprotective effect of L-serine may be partly due to an increase in rCBF in the ischemic brain through a vasodilating action mediated by SK_Ca_ and IK_Ca_ channels.

D-serine is an important co-agonist for NMDA receptors at the glycine-binding site and necessary for glutamatergic neurotransmission in many neurophysiological processes [16]. Overstimulation of NMDA receptors causing neuronal damage contributes to many neuropathological conditions, including acute cerebral ischemia [23], [24]. Hardingham et al. have identified that the synaptic and extrasynaptic NMDA receptors have the opposite effects on cyclic adenosine monophosphate response element-binding protein function, gene regulation and neuronal survival [25]. Activation of the synaptic NMDA receptors has anti-apoptotic activity, whereas stimulation of the extrasynaptic NMDA receptors causes loss of mitochondrial membrane potential and cell death [25].

To exclude an involvement of D-serine-related mechanisms in the neuroprotective effect of L-serine, the concentrations of L-serine and D-serine in the ischemic cortex and blood were measured. After intraperitoneal injection of L-serine, the blood level increased rapidly; after a 1 h delay the cortical level of L-serine in the ischemic side also reached a peak that was several times higher than the control. However, L-serine levels in the cortex of normal rats were not significantly elevated. The blood-brain barrier may be more permeable to L-serine under ischemic conditions. Therefore, with the help of serine racemase, D-serine levels in the ischemic cortex was elevated after injection of L-serine, although the peak level was reached with an additional 4 h delay and the amplitude was smaller than that seen with L-serine. These results are consistent with previous reports [26], [27]. However, we found that pretreatment of AOAA did not affect the recovery of neurological behaviors and the infarct volume of the brain in pMCAO rats treated with L-serine, suggesting that conversion of L-serine into D-serine by serine racemase in the brain may be not involved in the neuroprotective effect of L-serine.

**Figure 9:**
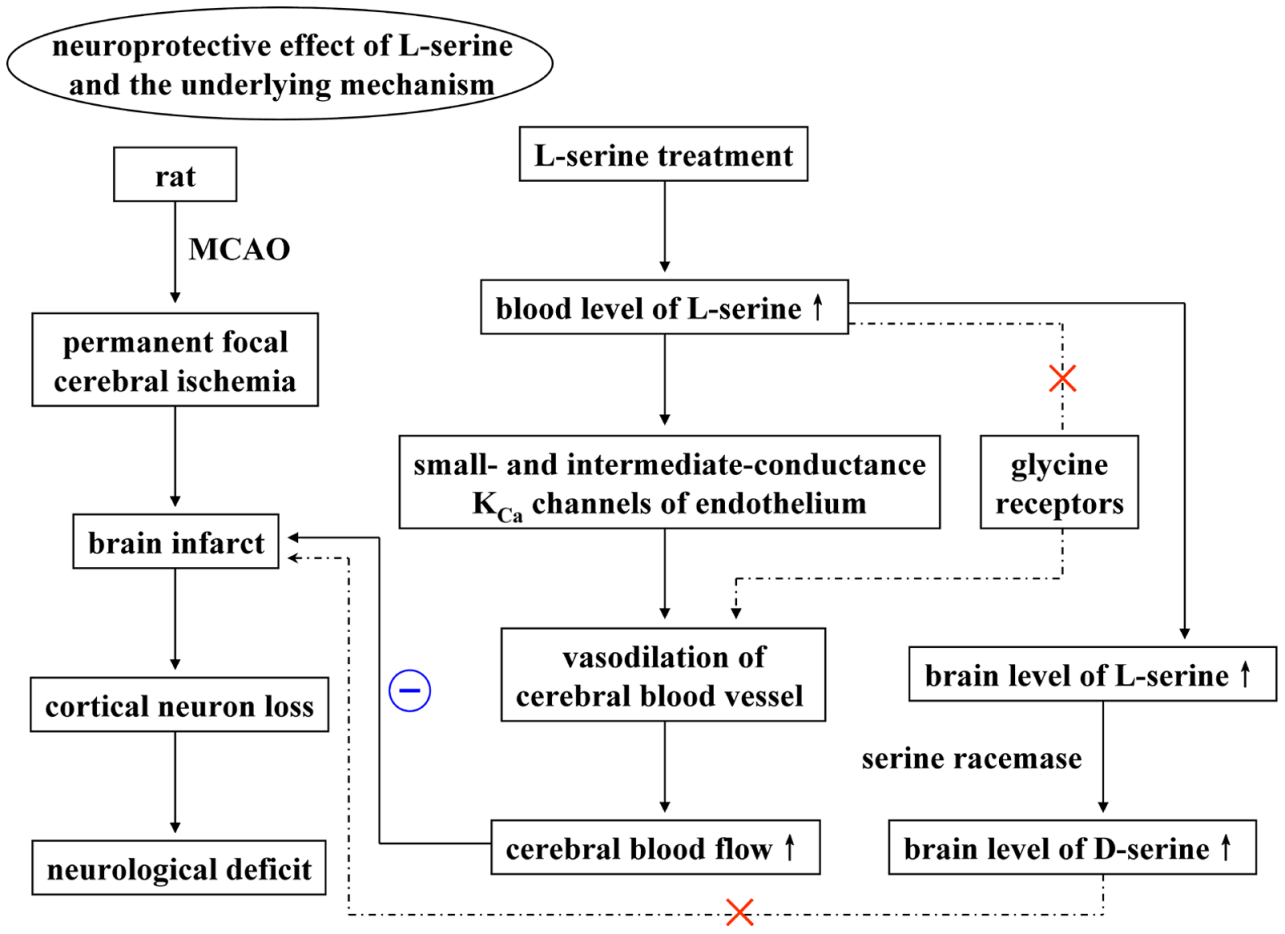
Graphical abstract. A flow diagram to reveal the potential mechanisms of the neuroprotective effect of L-serine treatment in rats after pMCAO.

In conclusion, L-serine treatment in pMCAO rats exerted a neuroprotective effect on the ischemic brain, at least partly due to an increase in rCBF through vasodilation mediated by the SK_Ca_ and IK_Ca_ channels on cerebral blood vessel endothelial cells. Activation of glycine receptors was not involved in this vasodilating action of L-serine, and neither was the neuroprotective effect of L-serine related to the conversion of L-serine into D-serine by serine racemase (Fig. 9).

## Supporting Information

Figure S1 **L-serine increased rCBF of the ischemic brain of mice after pMCAO.** (A) Examples of changes in the cerebral blood flow of mice in vehicle and L-serine groups. (B) Mean values of rCBF before and after MCAO. **P<0.01, vs. vehicle group. Young male C57BL/6 J mice (25–30 g body weight) were obtained from the Laboratory Animal Center, Chinese Academy of Sciences (Shanghai, China). The animals were housed in groups (4–5 per cage) with food and water available ad libitum, in a temperature- and humidity-controlled animal facility with a 12-h light/dark cycle. Mice were anesthetized with 1.5% isoflurane in 30% O_2_/68.5% N_2_O mixture under spontaneous breathing. With the mouse in a prone position fixed in a head holder (SG-4N, Narishige Co., Ltd.), the scalp was shaved and cut meticulously with a surgical knife to expose the thin skull over the bilateral cerebral and cerebellar hemispheres without causing brain trauma. The baseline CBF values were recorded for 5 minute by using the laser speckle technique (Stetler et al., 2012). Briefly, a CCD camera (PeriCam PSI System; Perimed) was positioned above the head, and a laser diode (785 nm) illuminated the intact skull surface to allow penetration of the laser in a diffuse manner through the brain. Speckle contrast (defined as the ratio of the SD of pixel intensity to the mean pixel intensity) was used to measure CBF as it is derived from the speckle visibility relative to the velocity of the light-scattering particles (blood). This was then converted to correlation time values, which are inversely and linearly proportional to the mean blood velocity. The mouse was then placed supine, and ischemia was induced. The rectal temperature was controlled at 37.0±0.5°C during surgery with a feedback regulated heating pad. After exposing the right carotid artery, a 5-0 silk suture was advanced into the internal carotid artery 12 mm from the lumen of the external carotid artery. The ipsilateral common carotid artery was occluded with a small surgical clip immediately after suture blockade. The animals underwent MCA occlusion permanently. L-serine of 268 mg/kg was intraperitoneally injected 30 min after pMCAO. Laser speckle perfusion images were obtained every 7.5 s, continuing throughout 90 min after pMCAO. CBF was analyzed two dimensionally over time by quantifying the area of infarct. The relative CBF was then reported as the ratio of baseline for each animal. Student’s *t*-test was used for the statistical analysis of results from vehicle group and L-serine treated group (6 mice for each group).
(TIF)
