# Supplementary figures and images for: Improvement in Regional CBF by L-Serine Contributes to Its Neuroprotective Effect in Rats after Focal Cerebral Ischemia
Source: PLoS One. 2013 Jun 25;8(6):e67044. doi: 10.1371/journal.pone.0067044 (PMC3692549; doi:10.1371/journal.pone.0067044)

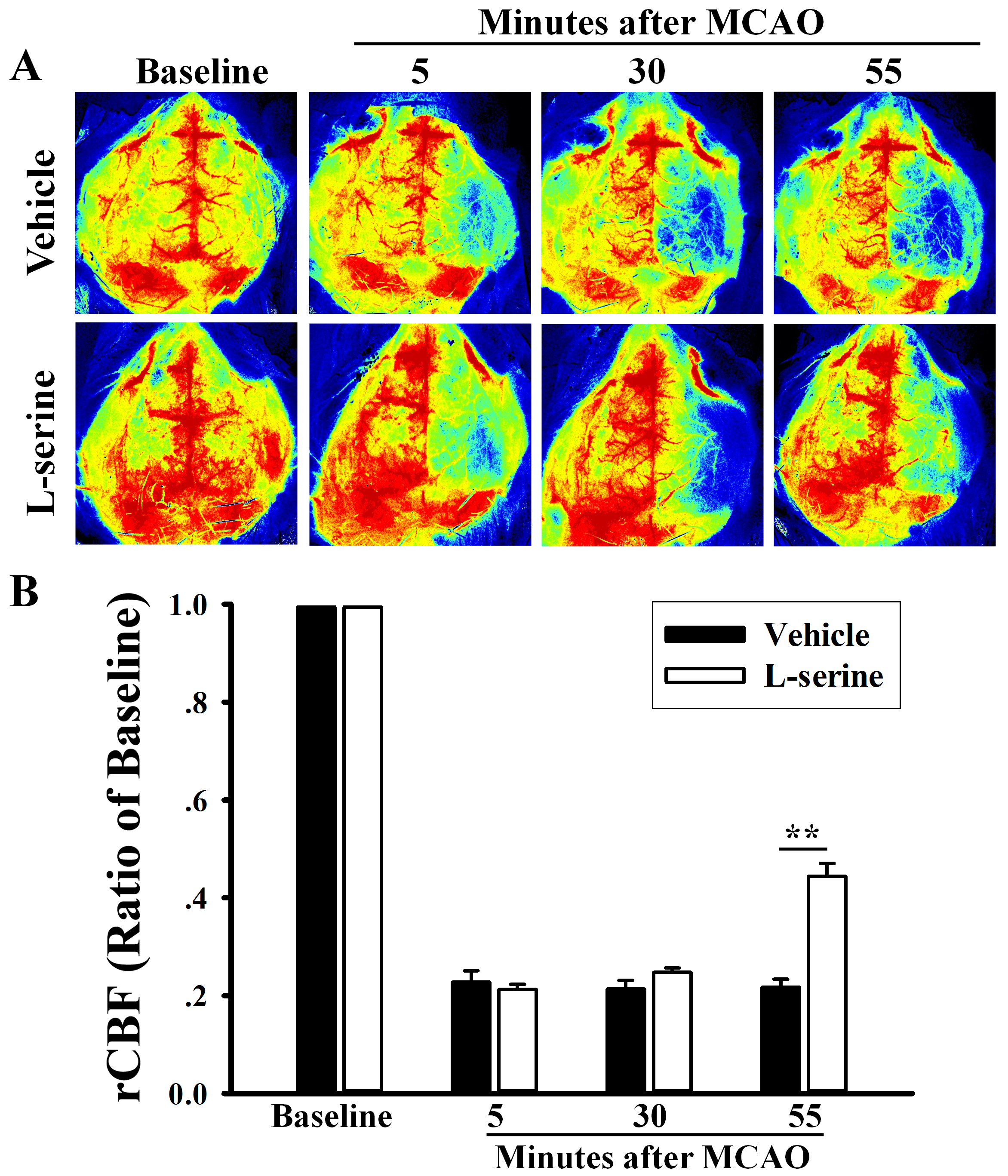

Supplement: Figure S1 — L-serine increased rCBF of the ischemic brain of mice after pMCAO. (A) Examples of changes in the cerebral blood flow of mice in vehicle and L-serine groups. (B) Mean values of rCBF before and after MCAO. **P<0.01, vs. vehicle group. Young male C57BL/6 J mice (25–30 g body weight) were obtained from the Laboratory Animal Center, Chinese Academy of Sciences (Shanghai, China). The animals were housed in groups (4–5 per cage) with food and water available ad libitum, in a temperature- and humidity-controlled animal facility with a 12-h light/dark cycle. Mice were anesthetized with 1.5% isoflurane in 30% O2/68.5% N2O mixture under spontaneous breathing. With the mouse in a prone position fixed in a head holder (SG-4N, Narishige Co., Ltd.), the scalp was shaved and cut meticulously with a surgical knife to expose the thin skull over the bilateral cerebral and cerebellar hemispheres without causing brain trauma. The baseline CBF values were recorded for 5 minute by using the laser speckle technique (Stetler et al., 2012). Briefly, a CCD camera (PeriCam PSI System; Perimed) was positioned above the head, and a laser diode (785 nm) illuminated the intact skull surface to allow penetration of the laser in a diffuse manner through the brain. Speckle contrast (defined as the ratio of the SD of pixel intensity to the mean pixel intensity) was used to measure CBF as it is derived from the speckle visibility relative to the velocity of the light-scattering particles (blood). This was then converted to correlation time values, which are inversely and linearly proportional to the mean blood velocity. The mouse was then placed supine, and ischemia was induced. The rectal temperature was controlled at 37.0±0.5°C during surgery with a feedback regulated heating pad. After exposing the right carotid artery, a 5-0 silk suture was advanced into the internal carotid artery 12 mm from the lumen of the external carotid artery. The ipsilateral common carotid artery was occluded with a small [file pone.0067044.s001.tif]
